# Modulation of instrumental responding by a conditioned threat stimulus requires lateral and central amygdala

**DOI:** 10.3389/fnbeh.2015.00293

**Published:** 2015-10-30

**Authors:** Vincent D. Campese, Rosemary Gonzaga, Justin M. Moscarello, Joseph E. LeDoux

**Affiliations:** ^1^Center for Neural Science, New York UniversityNew York, NY, USA; ^2^Emotional Brain Institute, Nathan Kline Institute for Psychiatric ResearchOrangeburg, NY, USA

**Keywords:** Pavlovian, instrumental, suppression, rat, motivation

## Abstract

Two studies explored the role of the amygdala in response modulation by an aversive conditioned stimulus (CS) in rats. Experiment 1 investigated the role of amygdala circuitry in conditioned suppression using a paradigm in which licking for sucrose was inhibited by a tone CS that had been previously paired with footshock. Electrolytic lesions of the lateral amygdala (LA) impaired suppression relative to sham-operated animals, and produced the same pattern of results when applied to central amygdala. In addition, disconnection of the lateral and central amygdala, by unilateral lesion of each on opposite sides of the brain, also impaired suppression relative to control subjects that received lesions of both areas on the same side. In each case, lesions were placed following Pavlovian conditioning and instrumental training, but before testing. This procedure produced within-subjects measures of the effects of lesion on freezing and between-group comparisons for the effects on suppression. Experiment 2 extended this analysis to a task where an aversive CS suppressed shuttling responses that had been previously food reinforced and also found effects of bilateral lesions of the central amygdala in a pre-post design. Together, these studies demonstrate that connections between the lateral and central amygdala constitute a serial circuit involved in processing aversive Pavlovian stimuli, and add to a growing body of findings implicating central amygdala in the modulation of instrumental behavior.

## Introduction

Pavlovian conditioned stimuli (CS; e.g., tone) produce defensive behaviors such as freezing after being paired with an aversive unconditioned stimulus (US; e.g., footshock; Pavlov, 1927). This form of learning depends on neural connections in the amygdala (LeDoux, 2000; Goosens and Maren, 2001; Fanselow and Poulos, 2005; Maren, 2005; Herry and Johansen, 2014; Lüthi and Lüscher, 2014; Janak and Tye, 2015). While the term Pavlovian fear conditioning is commonly used to describe this procedure, it implies that the subjective mental state of fear is being acquired. We have recently argued that the term Pavlovian threat conditioning (PTC) is preferable (and parsimonious) since the implication is that the stimulus is acquiring a new meaning (LeDoux, 2014, 2015).

In addition to eliciting defensive behaviors, a Pavlovian CS can also influence ongoing instrumental behaviors, and there is evidence that these modulatory processes depend on the amygdala as well. For example, the capacity for an aversive Pavlovian CS to augment footshock avoidance behaviors (e.g., shuttling in a two-way chamber: see Campese et al., 2013; and also, Bolles and Popp, 1964; Rescorla and LoLordo, 1965; Rescorla, 1968; Weisman and Litner, 1969; Overmier and Payne, 1971; Overmier and Brackbill, 1977; Patterson and Overmier, 1981) is impaired following lateral (LA), central (CeA) and medial (MeA) but not basal (BA) amygdala lesions (Campese et al., 2014; McCue et al., 2014). However, the role of amygdala circuitry is less clear in conditioned suppression, where the aversive CS reduces rather than facilitates behavior motivated by appetitive (or food based) reinforcement (Estes and Skinner, 1941; Estes, 1943). Existing studies of the effects of amygdala lesions on conditioned suppression have found contradictory results, due perhaps to differences in experimental design (e.g., few vs. many training trials; testing suppression in the same session as conducting CS-US pairings). Some other complications include the use of complex tasks in which suppression was but one of the components, or response type being measured (e.g., lever press, licking, wheel running), and areas of the amygdala targeted (LeDoux et al., 1988; Balleine and Killcross, 2006; McDannald and Galarce, 2011; Elrich et al., 2012). For example, studies have repeatedly shown that conditioned suppression requires the CeA (Killcross et al., 1997; Lee et al., 2005), but there have been inconsistent findings regarding the importance of lateral areas of the amygdala (e.g., LA and BA) in conditioned suppression (LeDoux et al., 1990; Killcross et al., 1997; Cardinal et al., 2002; Lee et al., 2005; Elrich et al., 2012; also see Fernando et al., 2013).

The current project sought to resolve discrepancies in the literature by examining the role of discrete amygdala nuclei in conditioned suppression of simple and naturally occurring behaviors by an aversive CS conditioned with a small number of CS-US parings. Using licking for sucrose reward, Experiment 1 evaluated the effects of bilateral LA, CeA and contralateral LA-CeA disconnection lesions on conditioned suppression by a shock paired CS. Conditioned freezing was also tested for each subject before and after surgery. Since LA lesions are known to disrupt freezing to the CS, it was expected that lesioned subjects would show both impaired freezing and conditioned suppression compared to sham subjects. Furthermore, it is well established that CeA lesions disrupt conditioned freezing (LeDoux et al., 1988; Collins and Paré, 1999; Amorapanth et al., 2000; LeDoux, 2000; Maren, 2005). Using lever press as the instrumental response, several studies have also demonstrated that conditioned suppression requires the CeA (Killcross et al., 1997; Amorapanth et al., 1999; Lee et al., 2005; Petrovich et al., 2009; McDannald, 2010). Because of the well established role of the CeA in freezing and suppression of other instrumental behaviors such as lever press, we expected that CeA lesions would disrupt freezing and impair conditioned suppression of instrumental licking similarly to LA lesions and also tested this in Experiment 1. Finally, while we expected to find that LA and CeA are both required for normal CS-elicited freezing and conditioned suppression, that result would not definitively demonstrate serial processing between these structures in these behaviors. Therefore, we also compared conditioned freezing and suppression in subjects with contralateral electrolytic LA and CeA lesions (i.e., disconnections) to control subjects. These controls received ipsilateral LA-CeA lesions on the same side of the brain. Due to the preservation of communication between LA and CeA in other the hemisphere in the ipsilateral group, we expected these subjects would show normal freezing and conditioned suppression. Contralaterally lesioned (or disconnection) subjects were expected to show impairments in both measures as a result of disconnecting LA from CeA.

Experiment 2 extended the analysis to a within-subjects task where shuttling for food reward was the instrumental response. In recent studies involving aversive instrumental avoidance we have used shuttling in a two-compartment chamber as the instrumental behavior (Choi et al., 2010; Lázaro-Muñoz et al., 2010; Campese et al., 2013, 2014; Moscarello and LeDoux, 2013; Ramirez et al., 2015). This is because behaviors that more closely approximate species-specific defensive responses (e.g., fleeing; Bolles, 1979) are more readily trained as avoidance responses than are other more artificial behaviors (e.g., lever press). One of the avoidance related tasks we have been studying is closely related to conditioned suppression both procedurally and also in terms of the underlying substrate. Essentially the inverse of conditioned suppression, conditioned facilitation (or PIT: aversive Pavlovian-to-instrumental transfer), involves the shock paired CS increasing ongoing footshock motivated shuttling (see Campese et al., 2013). This effect of the CS also depends on LA and CeA (Campese et al., 2014), begging the question of how the CeA can produce both increases and decreases in ongoing behavior when a CS is presented.

In order to compare CeA control of facilitation and suppression more effectively, we developed a conditioned suppression task that also uses shuttling as the instrumental response. In this task, rats shuttle in a two-compartment chamber to retrieve food on opposite ends of the context. The aversive CS is then presented over this behavior and its suppressive effect on shuttling rate is observed. Following conditioned suppression testing, subjects underwent the conditioned facilitation task (see procedures below and also Campese et al., 2013), providing within-subjects comparison of suppression and facilitation of shuttling behavior by the same CS in different tests. Furthermore, the importance of CeA in these modulatory behaviors was evaluated using lesions.

## Materials and Methods

### Subjects

For Experiment 1, 83 male Sprague-Dawley rats (Hilltop Lab Animals, Scottsdale, PA, USA) took part in the LA groups. Thirty-four were used in the CeA groups and twenty-three in the disconnection groups. Subjects weighed approximately 275 g at the start of the experimentation. Rats were single housed in standard Plexiglas cages on a 12:12 h light:dark cycle. All experimentation was conducted during the light phase. Subjects had free access to food and water while in their home cages, which were lined with paper bedding; subjects in Experiment 1 were never deprived of food or water. Animal care and housing was in accordance with Institutional Animal Care and Usage Committee (IACUC) policies and met the current standards of the Association for Assessment and Accreditation of Laboratory Animal Care International (AAALAC). All experimental protocols were approved by the University Animal Welfare Committee (UAWC) at New York University. Four rats took part in Experiment 2. These subjects were housed and cared for as described above. Except that during the unsignaled approach and conditioned suppression phases described below, these animals were food restricted. Subjects were given ad-lib access to food prior to approach training and 24 h prior to the start of training had their food removed. Supplemental rations were then provided at the end of everyday during these phases.

### Apparatus

For Experiment 1, Context a was a standard conditioning chamber manufactured by Coulbourn (Whitehall PA: Model No. H10-11R-TC). The chamber had a solid plastic floor and was scented with peppermint soap in the waste pan (Dr. Bronner’s magic soaps: Vista, CA, USA). The chamber was equipped with an 8-Ohm speaker (also manufactured by Coulbourn, Model No. H12-01R) and a licking spout in a recessed magazine. Context a was identical to context a except that no magazine module was present and the floors were stainless-steel parallel rods that carried the 0.7 mA footshock (Model No. H10-11R-TC-SF) produced by a precision shocker (Model No. H13-15) also manufactured by Coulbourn. There were also black and white stripes on the clear plastic walls. Context c was identical to context a except that citrus scented soap (also Dr. Bronner brand) was used and a checkered pattern was on the wall. Additionally there was no magazine module and the floor was made of thin crosswire patterned steel. All chambers were controlled using Graphic State 3 (Acimetrics, also by Coulbourn). All contexts were equipped with a programable tone generator (Model No. A12-33) and were housed in sound and light attenuating shells (Model No. H10-24A) also Coulbourn brand.

For Experiment 2, Context a was a standard Coulbourn chamber (as described for Experiment 1 above) with no soap scent, patterns or lick magazines. Context b was a two-way shuttle box manufactured by Coulbourn (Model no H10-11R-SC). Stainless steel grid floors delivered footshock during the avoidance phase described below and had speakers to present the CS: these devices were also from Coulbourn and were identical to those described in Experiment 1. Chambers were housed in sound and light attenuating cabinets. Context c was a two-way shuttle box assembled from white Plexiglas by hand and identical in size to Context b. The chamber had a food magazine and pellet (Testdiet, 40 mg) dispenser manufactured by Coulbourn (Model Nos. H20-94, H14-23R) on each end and was equipped with the Coulbourn speaker to present the 5 kHz tone CS.

## Procedure

### Experiment 1

#### Lick-Training

In context a, subjects were trained to lick a spout for a 20% sucrose solution; each lick produced a 0.3 mL volume reward. There were a total of six lick-training sessions, one per day, and the sessions were 1 h in duration.

#### Pavlovian Threat Conditioning (PTC)

The day after lick-training was completed, subjects were placed in context b and given 15 min to acclimate. There were then three 30 s tone conditioned stimulus (CS) presentations that coterminated with a 1 s 0.7 mA footshock US. These trials were separated by an average 180 s intertrial interval (ITI).

#### Pre-Lesion Test for CS Freezing

Twenty-four hours after PTC, subjects were placed in context c, and after a 5 min baseline period presented with two nonreinforced trials of the 30 s tone CS separated by a 3 min ITI.

#### Surgery

In the 3 days following the test for CS freezing, subjects received either LA electrolytic lesions or sham control surgeries. Subjects were anesthetized with ketamine and xylazine (i.p., 100 mg/kg; 6.0 mg/kg, Vedco). Once sedated, subjects’ heads were shaved and the rat was placed in a stereotaxic apparatus (David Kopf Instruments, Tujunga, CA, USA). An incision was made in the skin above the dorsal surface of the skull and tissue was retracted to reveal the landmarks bregma and lambda. Once these landmarks were adjusted to the same dorsal-ventral value, the electrode tip was placed on bregma and the unit was zeroed. Subjects received 10 s of 0.5mA current applied at the following three sites through holes drilled in the skull, for LA, AP-4.0 ML ± 5.5 DV-8.85, AP-3.2 ML ± 5.3 DV-8.85, AP-2.4 ML ± 5.1 DV-8.85 (coordinates in mm relative to Bregma). For CeA the three target sites were AP-3.3 ML ± 4.4 DV-8.9, AP-2.7 ML ± 4.2 DV-9.0, AP-2.1 ML ± 4.0 DV-9.1. For disconnections, the surgical objective was a functional disconnection of the LA and CeA. Therefore, subjects in the experimental condition received contralateral targeting of these structures. There were no sham subjects; instead control groups received ipsilateral targeting of these structures. The hemisphere targeting LA-CeA for ipsilateral and contralateral lesions was counterbalanced. The target sites and surgical parameters per structure were the same as used in for bilateral and disconnection surgeries. A monopolar stainless steel electrode was used (David Kopf Instruments, Tujunga, CA, USA: NE-300X for CeA and SNEX-300X for LA: 12 KΩ) and current from a Grass Instruments DC current generator (Model No D.C.LM5A) was applied. Control subjects had the electrode lowered to the sites, but no current was applied. Once these sites were treated, the head was sutured and the subject returned to the home cage for recovery.

#### Post-Lesion Test for CS Freezing

Two-weeks after surgery, subjects were returned to context c and again tested for freezing to the CS. This session was identical to the pre-lesion test.

#### Lick-Training Reminder Session

Twenty-four hours later, subjects were returned to context a and given an additional 1 h session of lick-training. This session was conducted identically to the previous lick-training sessions.

#### Test for Conditioned Suppression

The day following the reminder lick-training session, subjects were returned to context a and tested for conditioned suppression of licking by the tone CS. Following a 30 s baseline, ten lick responses were required to initiate the first trial. The first trial was comprised of a 20 s pre CS period followed by a 20 s presentation of the CS. A variable ITI ranging from 1–4 min separated the 10 trials. Following each ITI, ten responses were required to initiate the next trial.

#### Perfusion for Histological Analysis

Subjects were sacrificed with an overdose of chloral hydrate, transcardially exsanguinated with isotonic saline and perfused with 10% formalin. Brains were extracted, stored in formalin and cut into 40 μm sections on a microtome. The sections were stained with cresyl violet and evaluated for damage to the target structure under a light microscope. Only lesions that damaged at least 40% of the target structure and no more than 20% of surrounding structures were included (see Figure 4). These evaluation were made using Paxinos and Watson (2013) as a reference. For LA lesions, an additional criterion was that the dorsal portion of the structure had to be damaged for inclusion, thus ensuring that LA lesion subjects were missing the amygdala component most crucial for PTC learning (Repa et al., 2001; Rosenkranz and Grace, 2002; Han et al., 2007, 2009).

### Experiment 2

#### PTC

Subjects underwent a PTC session in context a where after a 5 min baseline, three trials of a 30 s 5 kHz tone coterminated with a 1 s 0.7 mA footshock. These trials were separated by 180 s.

#### Unsignaled Approach (Food Motivated Shuttling)

For 5 days, subjects were trained to retrieve food from opposite ends of context c (unsignaled approach or USAP). Food (2-pellets) was available, contingent on a shuttle response, on alternating ends of the chamber every 5 s. Subjects had to shuttle to produce 30 reward deliveries on each side of the chamber in order to complete a session.

#### Test for Conditioned Suppression

Following USAP training subjects were returned to context c where after 15 shuttle responses, a 1 min tone CS was presented followed by another criteria period of 15 responses and then a second trial, but of a novel noise stimulus. This sequence was repeated an additional time so that each stimulus was tested twice.

#### Unsignaled Sidman Active Avoidance

Twenty four hours after testing, subjects began USAA. USAA was conducted in context b, where a 1 s 0.7 mA footshock was presented every 5 s unless a shuttle response was made. Each shuttle postponed the next shock by 30 s and was accompanied by a 0.3 s blink of the houselights. There were fifteen of these 25 min USAA sessions. One session was given per day, with five sessions per week.

#### Test for Conditioned Facilitation

The day following completion of the USAA phase, subjects were tested for conditioned facilitation or Pavlovian-to-instrumental transfer (PIT) in context b. There were two tests, one each day for two consecutive days. During these tests subjects shuttled under extinction for a fixed baseline period of 15 min. At this point response rates were individually monitored and the CS presented to each animal separately when the subject’s response rate fell below two responses per minute for two full minutes. The CS then remained on until ten responses were made.

#### Surgery

Subjects received electrolytic lesions of the CeA following PIT tests. Surgeries were done as described above for CeA lesion groups except that a different current generator was used to produce damage to CeA (model 53500, Ugo Basile, Italy). All subjects were lesioned.

#### Post-Operative Tests for Suppression and Facilitation

Two-weeks after surgery, subjects were food restricted and 24 h later given another conditioned suppression test in context b. Following this test, animals were free fed and 1 week later given an additional session of PIT testing.

#### Perfusions and Histology

Perfusion and histology were done as described above, except that sections were stained with thionin.

## Results

### Experiment 1

As noted earlier, the roles of LA and CeA in conditioned suppression have not been clearly indentified in the literature (LeDoux et al., 1988; Balleine and Killcross, 2006; McDannald and Galarce, 2011; Elrich et al., 2012). Therefore, the effects of LA, CeA and LA-CeA disconnection lesions on both suppression of licking responses and conditioned freezing were evaluated in this study. The effects of lesions on freezing were measured within-subjects, before and after treatment while the effect on conditioned suppression was measured between groups. The results of this experiment show that in addition to deficits in conditioned freezing, LA, CeA and a functional disconnection of these nuclei impaired conditioned suppression by an aversive CS.

**Figure 1:**
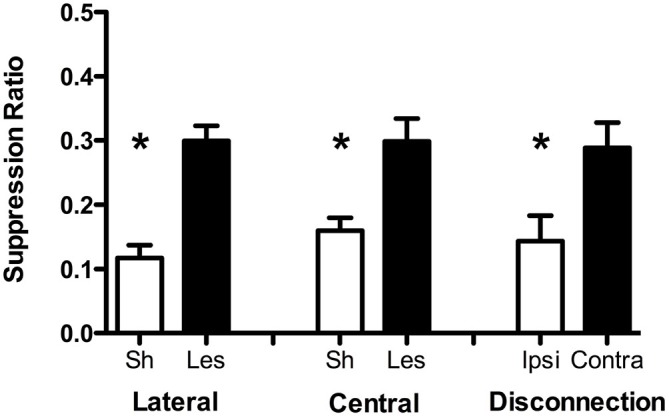
**Conditioned suppression test data from Experiment 1 for lateral (LA), CeA, and disconnection groups.** Data are presented in terms of the suppression ratio (*CSresponding/CSresponding + PreCSresponding*) for each group (Sh = sham, Les = lesion, Ipsi = ipsilateral, Contra = contralateral). Error bars reflect standard error of the mean. Asterisks indicate statistical significance (*p* < 0.05). Final sample sizes are as follow: LA sham = 27, LA lesion = 12, CeA sham = 14, CeA lesion = 10, ipsilateral = 7, contralateral = 8.

Data from the conditioned suppression test are presented in Figure 1 in terms of the suppression ratio (*CSresponding/CSresponding + PreCSresponding*) for each group. According to this measure, scores closer to zero reflect strong conditioned suppression. These data were analyzed with univariate analyses of variance (ANOVAs) with Surgery as a between-subjects factor for each group. In all cases these analyses produced significant effects of surgery (LA: *F*_(1, 36)_ = 25.44, *p* < 0.001, CeA: *F*_(1, 24)_ = 13.09, *p* = 0.002, Disconnection: *F*_(1, 15)_ = 6.72, *p* = 0.02). This analysis confirms the impression that LA or CeA lesions and disconnection of these structures impairs conditioned suppression relative to control subjects.

**Figure 2:**
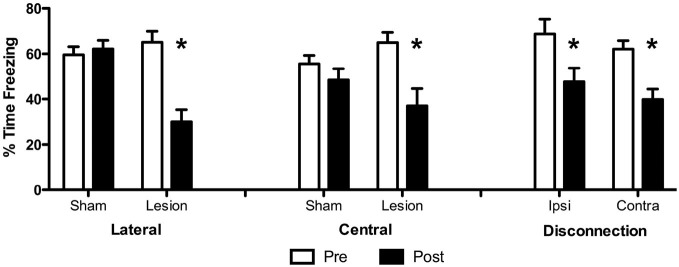
**Conditioned freezing data from Expeiment 1.** Data are presented in terms of percent time spent freezing to the conditioned stimulus (CS) before and after surgery for each group. *p < 0.05.

Freezing data from the pre and post surgical probe tests are presented in Figure 2 in terms of percent time freezing to the CS for each group. Freezing data for each group were analyzed with a 2 × 2 split-plot repeated-measures ANOVA including Test (pre or post) as the within-subjects factor and Surgery (sham or lesion) as the between-subjects factor. For LA and CeA groups these analyses confirmed the impression that lesions impaired CS-elicited freezing and produced significant main effects of Test (LA: *F*_(1, 34)_ = 33.5, *p* < 0.001, CeA: *F*_(1, 22)_ = 20.7, *p* < 0.001) and significant Test × Surgery interactions (LA: *F*_(1, 34)_ = 43.8, *p* < 0.001, CeA: *F*_(1, 22)_ = 7.3, *p* = 0.013). The main effect of Surgery was significant for LA (*F*_(1, 34)_ = 4.58, *p* < 0.05) but not CeA subjects (*F*_(1, 22)_ = 0.03, *p* = 0.87). For disconnection subjects, this analysis revealed that freezing was impaired by contralateral as well as ipsilateral lesions and produced a significant main effect of Test, *F*_(1, 13)_ = 32.13, *p* < 0.001. No significant effects were found among the Surgery factor, *F*_(1, 13)_ = 1.31, *p* = 0.3 or the Test × Surgery interaction, *F*_(1, 13)_ = 0.03, *p* = 0.87. These results show that while LA and CeA lesions impaired CS-elicited freezing compared to sham controls, both ipsilateral and contralateral (disconnection) LA-CeA lesions also impaired CS-elicited freezing.

### Experiment 2

**Figure 3:**
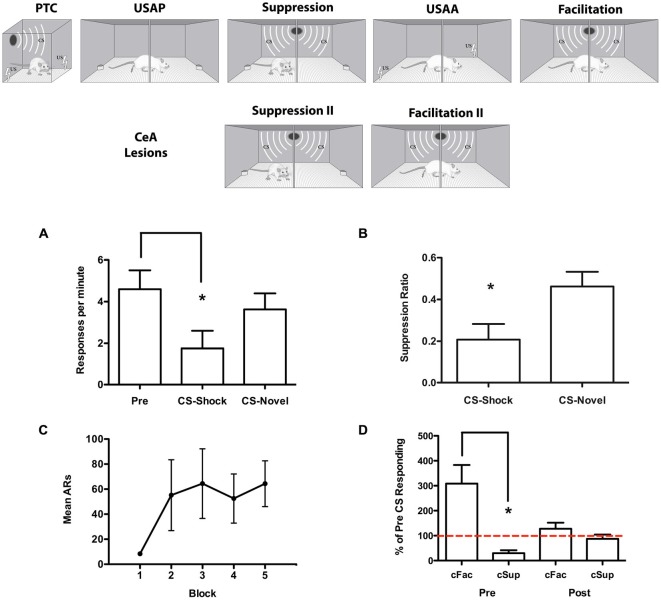
**Upper section:** Experimental design for the modulatory task used in Experiment 2. The Pavlovian threat conditioning (PTC) session took place on the first day of training. Subjects underwent the suppression (cSup) and then facilitation (cFac) components. USAP refers to unsignaled approach training and unsignaled sidman active avoidance (USAA) to USAA training phases where the two-way shuttle response was acquired in reward vs. avoidance procedures. After recovery from CeA lesions, additional tests were conducted. **Lower section:** Data collected for Experiment 2. Suppression test data are presented in **(A)** in terms of responses per minute during the Pre CS and CS periods and **(B)** in terms of the suppression ratio (*CSresponding/CSresponding + PreCSresponding*). USAA training data are presented in **(C)** for each three-session block in terms of mean avoidance responses per block. Data from the pre-surgical tests for conditioned facilitation and conditioned suppression are presented on the left of **(D)** and the post surgical tests on the right side. These data are presented in terms of percent of Pre CS responding for each test. *p < 0.05.

Recent findings suggest that CeA contributes to conditioned facilitation by an aversive CS (Campese et al., 2014). This result is seemingly at odds with the findings of Experiment 1 above where CeA was found to be important for the inverse effect of conditioned suppression. However, others studies have also found that opposing behaviors (i.e., freezing and fleeing) that depend on the same neural circuits (i.e., periacqueductal gray matter or PAG) can be contextually gated (see Kim et al., 2013). To test the dependence of both conditioned suppression and facilitation on CeA the sequence of training and testing procedures depicted in Figure 3 was used. This resulted in measures of both facilitation and suppression in each subject. Importantly, these effects were produced by the same CS which was tested in contexts where shuttling was either a food-reinforced response or a shock-avoidance response. The effects of CeA lesions were tested within-subjects as well by conducting pre and post-surgical tests in each context. The findings further suggest that CeA is critical for both suppression and facilitation of shuttling by an aversive CS. The design used in this study provides a powerful means of parsing the neural processes specific to each modulatory effect in a within-subjects procedure.

**Figure 4:**
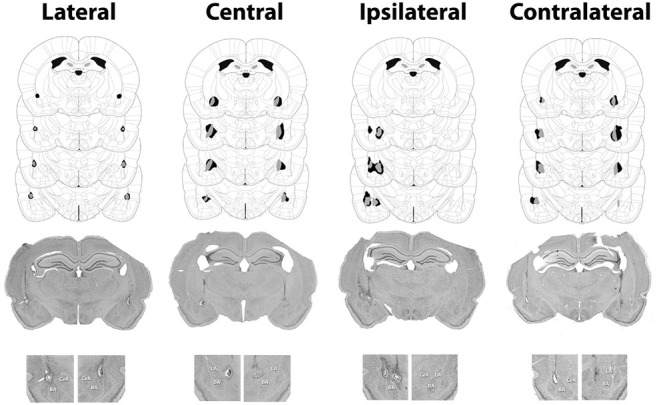
**Minimum (gray) and maximum (black) accepted lesions for subjects with bilateral LA or CeA lesions as well as ipsilateral or contralateral LA-CeA lesions.** Figures were adapted from Paxinos and Watson (2013) and profile damage 1.72, 2.16, 2.64 and 3.12 mm posterior to bregma. Representative images for each surgical condition are presented below the schematics with an enlargement of the amygdala region from each image presented below.

#### Test for Conditioned Suppression

Because no differences were found, Pre CS responding for each trial was averaged together. Responses during the stimuli were averaged as well and are presented below in Figure 3A in terms of responses per minute on the left and in terms of the suppression ratio on the right in Figure 3B. The responses per minute data from the suppression test were analyzed with a repeated measures analysis of variance including the within-subjects factors of Interval (pre or CS) and Stimulus (CS-shock or CS-novel), which found a significant effect of Interval, *F*_(1, 3)_ = 13.78, *p* = 0.03, and an Interval × Stimulus interaction, *F*_(1, 3)_ = 16.67, *p* = 0.03, with no main effect of Stimulus, *F*_(1, 3)_ = 3.57, *p* = 0.16, indicating suppression was only exhibited to the CS paired with shock. A *t*-test comparing suppression ratios for CS-shock and CS-novel, as expected, showed more suppression to CS-shock, *t*_(3)_ = 5.0, *p* = 0.02.

Data from the USAA phase are presented in Figure 3C. A repeated-measures analysis of variance found a significant effect of training session was obtained, *F*_(14, 42)_ = 2.91, *p* < 0.01, indicating that responding increased over sessions.* Test for conditioned facilitation*. PIT tests were conducted the day after USAA training ended and the data are presented in Figure 3D in terms of percent of Pre CS responding along side the suppression data to the tone presented in the same manner. In this form, any deviation from the 100% mark indicates modulation by the CS. These data suggest that the CS facilitates shuttling when the subject is avoiding shock, but suppresses shuttling when the subject is approaching food. This impression was confirmed with a *t*-test (*t*_(3)_ = 4.99, *p* = 0.015).* Post-operative tests for suppression and facilitation*. Following surgery, subjects showed no modulation of shuttling by the CS in either task. Data from the postoperative tests can be seen in Figure 3D and are presented in the same manner as described above. Clearly, the CS did not influence responding in either case, an impression, which was confirmed by a *t*-test that showed the deviation from baseline was minimally equal in the two tests, *t*_(3)_ = 1.72, *p* = 0.18. This analysis shows that the same tone CS enhanced shuttling in the shock context and suppressed it in the food context. Lesions of the CeA eliminated the capacity of the CS to modulate responding in either direction.

## Discussion

Experiment 1 found that lesions of LA and CeA disrupt conditioned suppression of instrumental behavior (licking). Control subjects showed normal freezing to the CS before and after surgery as well as normal conditioned suppression following surgery. LA and CeA lesions impaired both freezing and suppression. The CeA results replicate well-established findings regarding the effects of such lesions on conditioned freezing and suppression (Killcross et al., 1997; Amorapanth et al., 1999; Lee et al., 2005). More contentious have been conclusions regarding the contribution of LA. One study failed to find an effect of damage to the basolateral amydala (BLA), which includes LA, on suppression (Killcross et al., 1997). This study is often cited as evidence that CeA functions independent of LA in suppression (e.g., Cardinal et al., 2002; Balleine and Killcross, 2006; see Fernando et al., 2013). However, this study used large lesions of BLA that did not specifically target the dorsal LA, which has been shown to be the most crucial component of BLA for PTC (Repa et al., 2001; Rosenkranz and Grace, 2002; Han et al., 2007, 2009 for review see Maren, 2005; Johansen et al., 2011). In the current study, we ensured damage to this region in the LA lesion group, and found reliably that this impaired conditioned freezing and suppression relative to sham controls. This is consistent with results of other studies that have found that damage to LA disrupts conditioned suppression (LeDoux et al., 1990; Lee et al., 2005; Elrich et al., 2012). Because our training procedure did not include punishment of the lick response or any choice processes, the impairment we observed is likely attributable to suppression and not avoidance. We do not question reports suggesting that BLA participates tasks involving punishment (Jean-Richard-Dit-Bressel and McNally, 2015), choice (Killcross et al., 1997), and more generally assessing motivational value (Corbit and Balleine, 2005; Corbit et al., 2007). We used a minimally demanding behavioral response and relatively simple training requirements to measure suppression and produce the effect that we feel address the inconsistencies in these studies and demonstrate a clear role for these sub-regions. These more basic processes also need to be factored into conceptions of aversive instrumental behavior.

Furthermore, findings in Experiment 1 also provide strong evidence of the need for serial communication between these structures in conditioned suppression. While this has been repeatedly shown in standard aversive Pavlovian conditioning studies (Maren and Fanselow, 1996; Maren, 2001; Fanselow and Poulos, 2005; Jimenez and Maren, 2009; Ciocchi et al., 2010), it is not clear whether serial or parallel processing by the amygdala underlie the control over instrumental behaviors by Pavlovian cues (see Balleine and Killcross, 2006). In this study, surgical disconnection of the LA and CeA impaired freezing and conditioned suppression. Subjects with LA-CeA disconnections produced by contralateral lesions of these structures, showed impaired conditioned suppression compared to control subjects with ipsilateral lesions. Subjects with disconnections also showed impaired CS-elicited freezing as well following surgery in agreement with previous reports (Jimenez and Maren, 2009). However, while control subjects showed normal conditioned suppression, the ipsilateral LA and CeA lesions were also sufficient to produce an impairment in freezing to the CS. This is consistent with previous findings using ipsilateral lesions (LaBar and LeDoux, 1996; Jimenez and Maren, 2009). It should be noted that those studies compared the effects of these manipulations to sham groups, while the current study used a pre-post design including within-subjects analyses. This could potentially provide an explanation for the seemingly stronger effect of ipsilateral LA-CeA lesions seen here. Regardless of this, the dissociation of the effects of ipsilateral lesions on suppression vs. freezing found in Experiment 1 supports the hypothesis that response suppression and freezing are dissociable processes and not simply the result of competing responses. For example, it is well established that the CeA controls freezing responses through connections with the periacqueductal gray matter (PAG; see LeDoux et al., 1988; Amorapanth et al., 1999; Fanselow and Poulos, 2005; McDannald, 2010). In a study directly addressing this issue, Amorapanth et al. (1999) showed that even when the competing response (i.e., freezing) is removed, suppression is still seen. PAG lesions eliminated conditioned freezing, but they did not influence conditioned suppression. Because CeA lesions impair suppression as well as freezing and freezing is not necessary for suppression, this suggests that CeA produces a cessation of instrumental responding that is independent from the conditioned freezing it also controls. While our study was not designed to test this, the finding in the ipsilateral control condition is relevant to this idea. In summary, these data together with earlier findings provide very strong evidence that normal conditioned suppression depends on the CS-US learning stored in the LA producing a change in ongoing behavior (i.e., response suppression) via the CeA and not simply by promoting a competing response.

Experiment 2 demonstrated conditioned suppression using a new task. In this task, the subject shuttles in a two-compartment chamber to retrieve food and when tested, the aversive CS reduces shuttling rates. Importantly, this effect depends on associative learning; an untrained stimulus did not influence response rates during the test for suppression. This task was designed to mirror the aversive conditioned facilitation (or aversive PIT) procedure we’ve developed (Campese et al., 2013). This was done because in addition to the task similarities between suppression and facilitation, they also appear to depend on a shared circuit. In aversive PIT, subjects shuttle to avoid footshock, and the CS enhances shuttling rates, an effect which also depends on LA and CeA (Campese et al., 2013, 2014). Subjects from Experiment 2 underwent training on this facilitation task after initial suppression testing was complete providing a within-subjects measure of both suppression and facilitation by the same CS. These subjects showed facilitation by the aversive CS when tested in the shock-motivated shuttling context and suppression when testing occurred in the food-motivated shuttling context. It was then shown in post-surgical tests, using these same subjects, that both modulatory effects of the CS were eliminated following CeA lesions, replicating findings where these tasks had been conducted separately (LeDoux et al., 1990; Killcross et al., 1997; Amorapanth et al., 1999; Cardinal et al., 2002; Lee et al., 2005; Elrich et al., 2012; Campese et al., 2014). It is an intriguing question as to how the CeA is capable of exerting opposing effects on the same physical response in the presence of the same CS. While the underlying neural mechanisms of suppression and facilitation are likely distinct in at least some ways, both require the CeA, suggesting that perhaps separate output pathways from CeA are likely responsible for these phenomena. For example facilitation but not suppression might require CeA-accumbens connections in order to exert control over motor pathways. Alternatively CeA-hypothalamus connections may be required due to the similarity of shuttling to prepared feeing behaviors. In a recent study, Kim et al. (2013) found that dorsal PAG stimulation led to activity burst behaviors (followed by freezing) during a standard PTC task but fleeing during a foraging task. This would suggest that the context (safety vs. danger) could, in some way, prime the valence of the output. This process could theoretically occur using the same circuits processing a shock paired CS, producing suppression in a safe context and facilitation in a dangerous one. Perhaps this is due to the proximity of the threat. For example, freezing and fleeing can be understood as being released at different proximities to predators/aversive events (Fanselow and Lester, 1988). That shock has occurred in the avoidance but not the food context may place the subject closer to the threat and release one behavior in preference of the other. Further studies are clearly needed to investigate the precise circuitry in the CeA that controls these opposing effects.

One limitation in the interpretation of these results is that electrolytic lesions were used. This technique damages fibers as well as cell bodies, and can produce effects that depend on areas other than the lesion site. The field is turning towards more selective tools in assessing the circuitry of conditioned suppression. Because the current studies used small and controlled lesions, these results provide an important contribution towards the understanding of the role of the amydala in conditioned suppression (Balleine and Killcross, 2006).

In summary, these data provide evidence that LA and CeA appear to work in sequence to produce freezing and conditioned suppression responses. It is important to acknowledge that these are separate processes (Amorapanth et al., 1999) and that modulation may rely on more complex circuitry. An aversive CS is capable of facilitating a shock-avoidance response, while in the same subject also suppressing an appetitive response with the same physical characteristics as the former. This suggests that the response modulation mechanism (likely based in CeA) somehow incorporates the motivational valence of the instrumental and Pavlovian outcomes in the process.

## Author Contributions

VC collected data, designed studies and wrote the manuscript. RG collected data. JMM designed studies (Experiment 1) and assisted in writing the manuscript. JEL designed studies and also wrote the manuscript.

## Conflict of Interest Statement

The authors declare that the research was conducted in the absence of any commercial or financial relationships that could be construed as a potential conflict of interest.
